# Protective signature of xanthohumol on cognitive function of APP/PS1 mice: a urine metabolomics approach by age

**DOI:** 10.3389/fphar.2024.1423060

**Published:** 2024-07-24

**Authors:** Wei Liu, Xiao Chen, Jing Zhao, Chen Yang, Guanqin Huang, Zhen Zhang, Jianjun Liu

**Affiliations:** ^1^ Shenzhen Center for Disease Control and Prevention, Shenzhen, China; ^2^ Shenzhen Key Laboratory of Modern Toxicology, Shenzhen Medical Key Discipline of Health Toxicology (2020-2024), Shenzhen Center for Disease Control and Prevention, Shenzhen, China

**Keywords:** xanthohumol, Alzheimer’s disease, neuroprotective effect, metabolomics, urine, mouse

## Abstract

Alzheimer’s disease (AD) has an increasing prevalence, complicated pathogenesis and no effective cure. Emerging evidences show that flavonoid compounds such as xanthohumol (Xn) could play an important role as a dietary supplement or traditional Chinese herbal medicine in the management of diseases such as AD. This study aims to analyze the target molecules of Xn in the prevention and treatment of AD, and its potential mechanism from the perspective of metabolites. APP/PS1 mice 2- and 6-months old were treated with Xn for 3 months, respectively, the younger animals to test for AD-like brain disease prevention and the older animals to address therapeutic effects on the disease. Memantine (Mem) was selected as positive control. Behavioral tests were performed to assess the course of cognitive function. Urine samples were collected and analyzed by high-performance liquid chromatography (HPLC) with tandem mass spectrometry (MS/MS) coupled with online Compound Discoverer software. Morris Water Maze (MWM) tests showed that Xn, like Mem, had a therapeutic but not a preventive effect on cognitive impairment. The expression levels of urinary metabolites appeared to show an opposite trend at different stages of Xn treatment, downregulated in the prevention phase while upregulated in the therapy phase. In addition, the metabolic mechanisms of Xn during preventive treatment were also different from that during therapeutic treatment. The signaling pathways metabolites nordiazepam and genistein were specifically regulated by Xn but not by Mem in the disease prevention stage. The signaling pathway metabolite ascorbic acid was specifically regulated by Xn in the therapeutic stage. In conclusion, dietary treatment with Xn altered the urinary metabolite profile at different stages of administration in APP/PS1 mice. The identified potential endogenous metabolic biomarkers and signal pathways open new avenues to investigate the pathogenesis and treatment of AD.

## 1 Introduction

Alzheimer’s disease (AD) is the most common form of dementia and may contribute to 60%–80% of dementia cases (Alzheimer’s Association Report, 2023). The clinical presentation of AD manifests in impaired speech expression, with deficits in learning and memory which gradually deteriorates from mild cognitive impairment (MCI) to severe cognitive impairment. At present, more than 50 million people worldwide are affected by AD, and the number is expected to exceed 150 million by 2050 (WHO, 2019). AD patients in China account for about a quarter of the total cases worldwide. AD not only seriously affects the quality of life of patients, but also brings a huge economic burden to society (Tahami Monfared et al., 2022).

Because of its prolonged incubation and complex pathogenesis, there is currently no plan to prevent, cure or change the progressive course of AD. The U.S. Food and Drug Administration (FDA) approved four drugs to treat the disease: donepezil, rivastigmine, galantamine, and memantine (Mem). The first three, as acetylcholinesterase inhibitors, alleviate AD symptoms by enhancing acetylcholine neurotransmission, while the fourth, a low-non-competitive NMDA receptor antagonist, alleviates cognitive impairment by reducing excitatory neurotoxicity (Scheltens et al., 2016). While drug treatment alleviate symptoms, they have no effect on the unrelenting downhill course of AD.

Natural products have recently attracted attention as potential therapeutic agents for AD (Liu et al., 2023; Yang et al., 2023). In particular, the female inflorescences of *Humulus lupulus* Linneus, commonly known as hops, have long been used in traditional medicine to treat many diseases (Lin et al., 2019). Xanthohumol (Xn), a unique prenylated chalcone, is a bioactive ingredient isolated from hops. Dietary Xn improves cognitive flexibility in young mice, but is ineffective in adjusting the age-related palmitoylation status of neuronal proteins in aged animals (Zamzow et al., 2014). Our previous behavioral studies showed that Xn reduced cognitive dysfunction in APP/PS1 mice and significantly regulated the composition and abundance of murine gut microbiota in young and old animals (Liu et al., 2022). Xn inhibited the Ca^2+^ calmodulin/PKA cascade by reducing Ca^2+^ inflow through acting on γ-aminobutyric acid receptors in rat hippocampal synapses resulting in suppression of glutamate release (Chang et al., 2016). In addition, Xn moderately inhibited the enzyme activity of acetylcholinesterase relative to that of galanthamine (Orhan et al., 2018). All these studies suggested that Xn may be a candidate drug molecule for the prevention and treatment of AD.

Metabolomics provides a novel approach for the drug discovery and potential biomarkers of diseases such as AD (Jiang Y et al., 2019). As the “terminus” of genome, transcriptome and proteome, the metabolome can more directly and accurately reflect the pathological and physiological state of organisms. While each biological fluid has its own unique metabolic footprint, urine is a key biological matrix in metabolite-profiling studies, and its collection is noninvasive (Kurbatova et al., 2020). A combination of nine urinary metabolites with an area under the curve value of 0.976 showed a significantly difference between 30 AD patients and 30 cognitively normal individuals (Zhang et al., 2022). Eleven urinary metabolites identified by a targeted, quantitative metabolic method were significantly altered in AD patients, with a sensitivity of 76%, specificity of 75%, and accuracy of 81% (Yilmaz et al., 2020). These observations raise the possibility that alterations in urinary metabolite signatures could serve as biomarkers for the detection and staging of AD.

The present study uses APP/PS1 double transgenic mice expressing a chimeric mouse/human amyloid precursor protein and a mutant human presenilin 1, both associated with early-onset AD. We employed a comprehensive non-targeted metabolomics analysis of urine samples of 2- and 6-month-old of APP/PS1 mice after Xn treatment was performed to find differential metabolites and the biological pathways associated with AD.

## 2 Materials and methods

### 2.1 Materials and reagents

Xn [CAS#6754-58-1, purity ≥97.0% (HPLC)] and corn oil were purchased from Aladdin Biochemical Technology Co., Ltd. (Shanghai, China). Memantine hydrochloride [CAS#41100-52-1, purity ≥98% (GC)] was obtained from Sigma-Aldrich, Inc. (Saint Louis, MO, United States). Methanol and formic acid, which were HPLC-grade, were obtained from Sigma-Aldrich (Shanghai) Trading Co., Ltd. (Shanghai, China). Water was prepared by purification systems (Millipore Co., Ltd. Billerica, MA, United States).

### 2.2 Animal models and Xn administration

APP695swe/PS1-dE9 (APP/PS1) mice were used in the study, C57BL/6 wild type (WT) mice of the same age were selected as control. Two-month-old mice (i.e. 60 days of age) and 6-month-old animals (180 days of age) were used to assess the effect of Xn treatment on disease prevention and treatment, respectively. Animals were treated by gavage with 5 mg/kg Xn (dissolved in corn oil) or corn oil alone (0.1 mL/10 g body weight) every other day for 90 days. Since poor pharmacokinetic properties, Xn is freshly prepared for use. The oral dosage and administration of Xn was selected based on a previous report (Rancán et al., 2017). Age-matched animals treated with 5 mg/kg memantine (Mem, dissolved in ultrapure water) served as positive controls. The preventive and therapeutic treatment groups comprised equal numbers of male and female mice consisting of 9–11 and 11–14 animals, respectively.

Animal treatment and care were performed in accordance with the principles of laboratory animal care (NIH publication No. 85-23, revised in 1985) and the regulations for animal care and use from the committee of experimental animal center at Shenzhen Center for Disease Control and Prevention (SZCDC) in Shenzhen, Guangdong province, China. This animal study (NO.2022019) was approved by the SZCDC Ethics Committee. Efforts were made to minimize animal suffering and reduce the number of mice used for experiments. Food and water were provided *ad libitum*.

### 2.3 Behavioral tests

Animals were subjected to an open field test (OFT) and the Morris water maze (MWM) test. OFT is a behavioral study used to evaluate the exploration behavior and tension of animals in an open environment, mainly reflecting the anxiety level (Kraeuter et al., 2019). The experimental device consisted of a square white plastic board with a bottom (50 cm × 50 cm) and a black plastic board with a height of 40 cm. The open field was divided into 16 small square areas, including 12 peripheral areas (yellow) and four central areas (blue). During the experiment, the whole device was illuminated with 80 lux light, and the mice moved freely in the open field, while a video analysis system (BW-OF302, Shanghai Bio-will Co., Ltd. China) automatically recorded activity for 5 min. The smaller the ratio of central time to total time and the smaller the ratio of central distance to total distance were taken as measures of higher degrees of anxiety.

The Morris water maze (MWM) test was used to evaluate spatial learning and memory, as described (Vorhees et al., 2006) and employed in our previous work (Liu et al., 2022). Briefly, the MWM test mainly includes two parts: place navigation experiment (training period) and spatial probe experiment (testing period). The training period is 5 days. The mean escape incubation period of 4 times per day was calculated to evaluate the spatial learning ability of animals. On the 7th day, the platform was removed and the space probe experiment was conducted. The observation time was 2 min. The system automatically recorded the time the mice reached the platform for the first time (spatial probe time), the number of times of platform crossing, the mouse swimming track and other parameters.

### 2.4 Urine metabolomics analysis

#### 2.4.1 Urine samples collection

Urine samples were collected from mice at 5 months of age (preventive treatment) and 9 months of age (therapeutic treatment), respectively. Metabolic-cage collection of urine samples was used in the study. After an overnight stay in the metabolic cage, urine was collected the next morning and stored in aliquots at −80°C for further analysis.

#### 2.4.2 Urine samples processing

Urine samples were processed as previously reported (Want et al., 2010). Briefly, samples were thawed on ice at 4°C for 30 min 200 μL methanol (pre-cooled in −80°C) was added to 50 μL urine and gently vortexed for 1 min for efficient extraction. Samples were incubated at room temperature for 10 min and then at −20°C overnight (16 h). Centrifugation was then performed at 14,000 g, 4°C for 20 min to remove particulates. Then, all supernatants were transferred to a new 1.5 mL EP tube for freeze drying (RVC 2-18 CD plus, Christ^®^, Germany). The samples were then redissolved in 100 μL sterile water and mixed by vortex for 1 min followed by centrifugation at 14,000 g for 20 min at 4°C. The supernatant was then taken for metabolite analysis. In addition, methanol samples (blank control) and mixed samples (QC control) were also treated. To obtain high quality data, equal urine samples from each group were mixed for quality control (QC) samples.

#### 2.4.3 Urine metabolic components determination by HPLC-MS/MS

HPLC-MS/MS analysis was performed via an Ultimate 3000 system (Thermo Fisher Scientific, CA, United States) equipped with a Q Exactive mass spectrometer (Thermo Fisher Scientific, CA, United States). This analysis was performed according to the manufacturer’s instructions and reported literature (Want et al., 2010; Yuan et al., 2012; Hao et al., 2018). Before every analysis, instrument calibration was performed using the Pierce LTQ ESI negative ion calibration solution (Thermo Fisher Scientific, United States) for negative mode. Mass accuracy was checked to be 0.5 ppm but was routinely below 2 ppm. An Atlantis^®^ dC 18 Column (5 μm, 2.1 mm × 150 mm, 100 Å, Waters, Milford, Massachusetts, United States) was used to separate analytes under 40°C. The mobile phase consisted of ultra-pure water containing 0.1% (V/V) formic acid (phase A) and methanol containing 0.1% (V/V) formic acid (phase B). The gradient with phase B was as follows: 0–1 min, 5%; 1–15 min, 5%–95%; 15–16 min, 95%–99%; 16–19 min, 99%; 19–19.2 min, 99%–5%; 19.2–25 min, 5%. Total run time was 25 min, pumping 0.3 mL/min with a column temperature of 55°C, acquiring in negative mode. The injection volumes were 2 μL. Data were obtained with the MS detector in the following parameters: full-scan mode (mass range, 70–1,050 Da), 5 ppm mass tolerance, 0.05 min retention time (RT) tolerance, signal to noise ratio threshold (1.5) and high resolution (70,000) with the data-dependent acquisition (dd-MS2).

#### 2.4.4 Metabonomics data analysis

Identified metabolic ions were then analyzed by Compound Discoverer (CD) software (version 3.0.0.294, ThermoFisher Scientific, San Jose, CA, United States) (Züllig et al., 2020). Raw data were first processed for baseline correction, retention time (RT) alignment, peak extraction and alignment, peak area normalization, metabolite alignment identification, etc. The processed data were then identified by comparing their retention time, mass value, and MS/MS fragmentation with the corresponding parameters in the CD software. Metabolite IDs were also confirmed with an in-house metabolite library and available metabolite standards by comparing their LC retention time, precursor m/z and MS/MS spectra. In addition, full-scan mass spectra of these metabolites were interpreted using biochemical databases including the ChemSpider pathways (http://www.chemspider.com/), the mzCloud pathways (https://www.mzcloud.org/), and the MetaboAnalyst online website (https://www.MetaboAnalyst.ca/). Metabolic pathway analysis function is available in the CD software where metabolite IDs were mapped into the Kyoto Encyclopedia of Genes and Genomes (KEGG, http://www.genome.jp/kegg/) pathways.

### 2.5 Bio-informatics analysis of differential metabolites

The metabolites of statistical significance were defined as *p* < 0.05 and annotation source had full match status in any source of predicted compositions search, ChemSpider, mzCloud, and Metabolika. The differential metabolites were defined as group areas had any value in any sample group and group CV% was less than 30 in any sample group on the basis of statistically significant metabolites. The significant metabolites and differential metabolites were then analyzed for visual bio-informatics analysis. Heat map, advanced volcano plot, principal component analysis (PCA) and KEGG pathway were plotted using the OmicStudio tools at https://www.omicstudio.cn/tool.

### 2.6 Statistical analysis

SPSS version 25.0 was used to analyze the data. One-way ANOVA was used to compare the mean between groups followed with the LSD-t test. Repeated measures ANOVA was used in the MWM test. Graphs were drawn via Graphpad Prism Software (version 8.2.1, La Jolla, CA, United States), and the data were presented as the mean ± standard error of mean (SEM). Mann Whitney Wilcoxon test was applied to measure the significance of each peak in the different groups, with results adjusted for multiple testing using false discovery rates (FDR) correction. Two-sided *p* values <0.05 had statistical significance.

## 3 Results

### 3.1 Xn improved the performance of cognitive function in APP/PS1 mice

**FIGURE 1:**
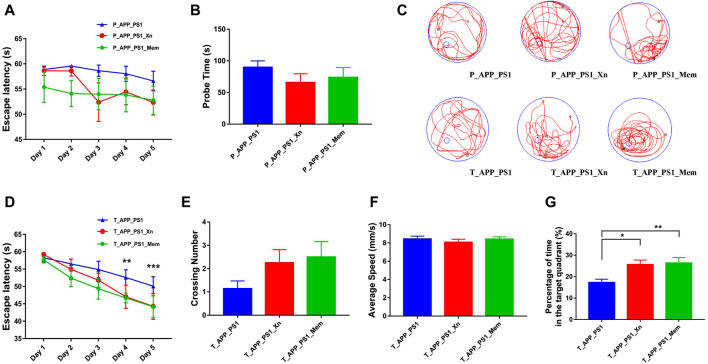
Xn and memantine improved the learning and memory ability in APP/PS1 mice. **(A)** Escape latency during platform trial test in the prevention experiment (n = 7–11 per group). **(B)** Probe time in the spatial probe test. **(C)** Representative tracing graphs. **(D)** Escape latency in the therapy experiment (n = 10–14 per group). **(E)** Crossing number in the spatial probe test. **(F)** Average speed. **(G)** Percentage of time spent in the target quadrant. *p < 0.05, **p < 0.01, ***p < 0.001. Error bar, standard error of mean (SEM).

The OFT was used to evaluate whether treatment with Xn (vs Mem) could improve anxiety and depression. The results showed no significant difference in time ratio and distance ratio (Supplementary Figure S1). Neither prophylactic nor therapeutic treatment with Xn or Mem showed a meaningful ability to reduce anxious behavior in APP/PS1 mice. Based on our previous study (Liu et al., 2022), the MWM test was used to compare the difference between Xn and Mem in improving the ability of spatial learning and memory (Figure 1). In the preventive experiments, with the increase of learning time, both Xn- and Mem-treated mice showed certain learning ability, and the space probe time was also shortened, but there was no significant difference (Figures 1A–C). In the therapeutic experiment, as the learning time increased, both the Xn- and Mem-treated mice showed better learning ability compared to drug-untreated APP/PS1 mice (Figure 1D). There was no significant change in the swimming speed in all mice, but the number of times that the Xn- and Mem-treated mice crossed the hidden platform was increased (Figures 1C,E,F). Moreover, mice treated with Xn or Mem spent significantly more time in the target quadrant (Figure 1G). In sum, Xn, like Mem, had a better therapeutic effect than preventive effect on cognitive impairment.

### 3.2 Xn altered the urinary metabolite profiles at different stages of administration

Compound Discoverer software was used to analyze endogenous metabolites in urine samples. A total of 11,917 metabolites was found in the preventive experiments, of which 60 compounds were statistically significant in the Xn-treatment group and 628 were statistically significant in the Mem treatment group compared with the AD model group (Supplementary Figures S2A, S2B). A total of 9,054 compounds was found in the therapeutic experiments, of which 96 compounds were statistically significant in the Xn-treatment group and 76 were statistically significant in the Mem treatment group compared with the AD model group (Supplementary Figures S2C, S2D). Interestingly, we found clear changes in urine metabolites treated at different stages: in the preventive stage, the metabolites of Xn treatment mainly showed a downward trend, while in the therapeutic stage, there was significant upregulation, with Mem treatment the more obvious.

**FIGURE 2:**
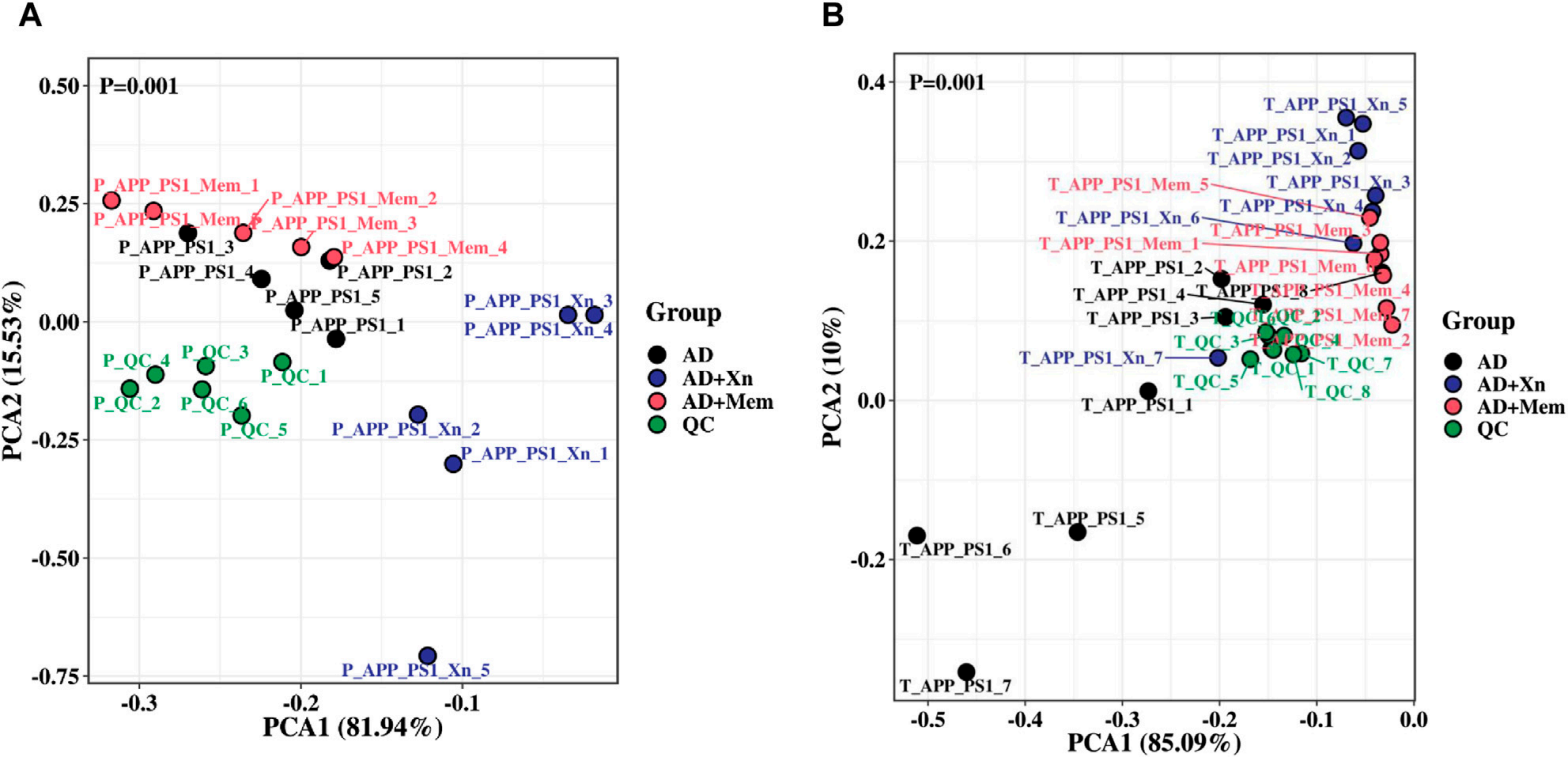
A two-dimensional PCA scores plot of urine samples and QCs obtained in different experimental stages. **(A)** PCA score plot indicating the separation among groups during preventive treatment and **(B)** the separation during therapeutic treatment. In the figure, closer samples had higher similarity, while different samples separated from each other.

**FIGURE 3:**
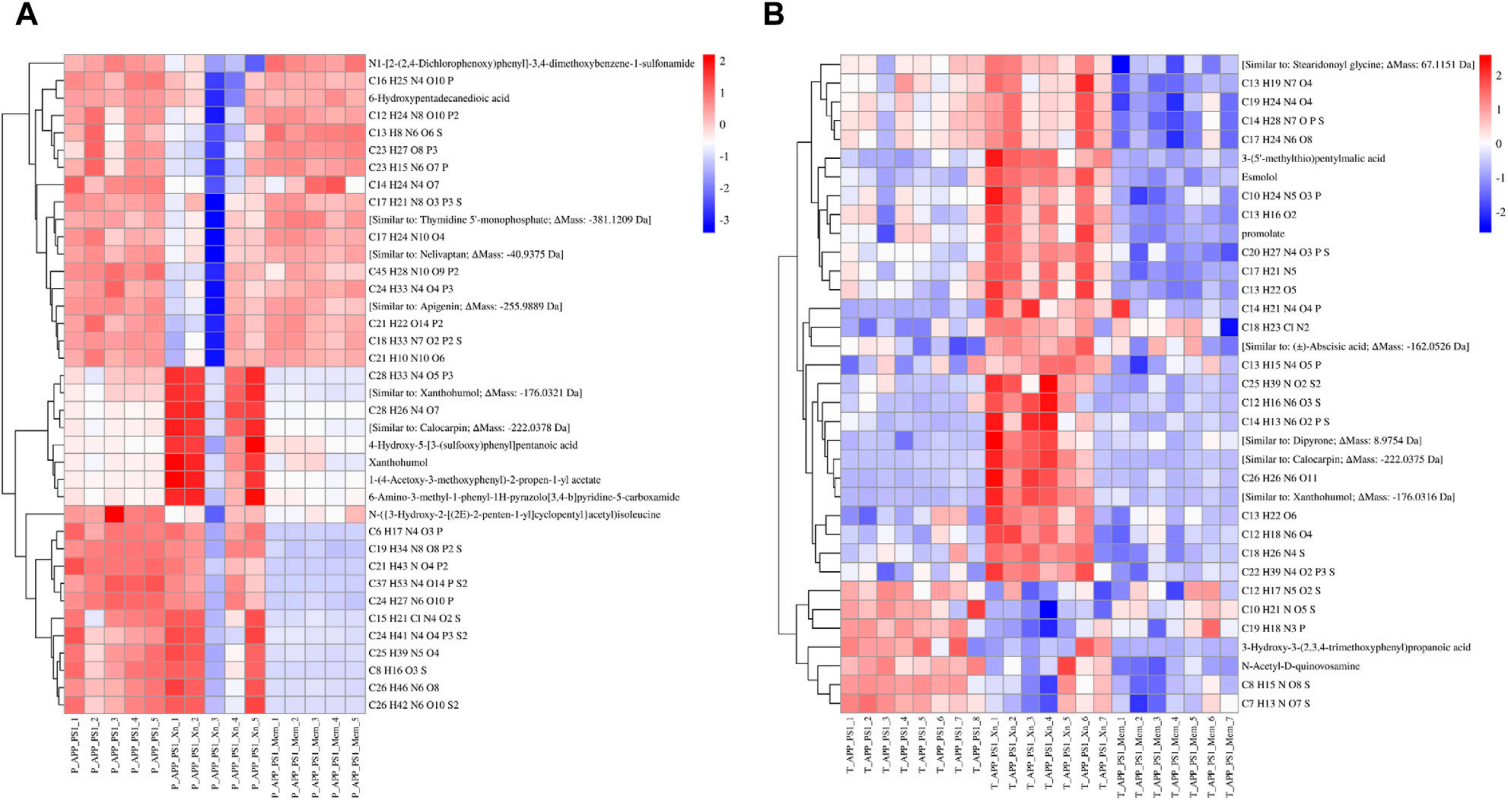
Heat map analysis of urinary differential metabolites among the groups. **(A,B)** showed the comparison results in the preventive and therapeutic treatment groups, respectively. The heat map was a more intuitive way to understand the trend of all the differential metabolites in each sample. Red indicates upregulated while blue indicates downregulated. The horizontal axis represents samples in different groups and the vertical axis represents differential metabolites. Each rectangle represents the relative amount of a particular compound in a particular sample.

To understand the overall stability of urine samples in each group and to search for differential metabolites, we then performed visual analysis. PCA results showed that, in both preventive and therapeutic treatment, the QC samples were close together, suggesting good stability and reliability (Figure 2). The AD mouse group, the Xn-treated group and the Mem-treated group could be separated from each other. Notably, results for the Mem-treated group was closer to that of the AD group in the preventive experiment while closer to the Xn-treated group in the therapeutic experiment. Based on the significant metabolites, we further screened 40 differential metabolites in the preventive experiments and 35 differential metabolites in the therapeutic experiments. Heat-map results showed that, the expression levels of differential metabolites were significantly different among the groups (Figure 3). Similarly, metabolite levels varied between Xn and Mem treatments at different stages.

### 3.3 Xn regulated the KEGG signal pathways of urinary metabolites

**FIGURE 4:**
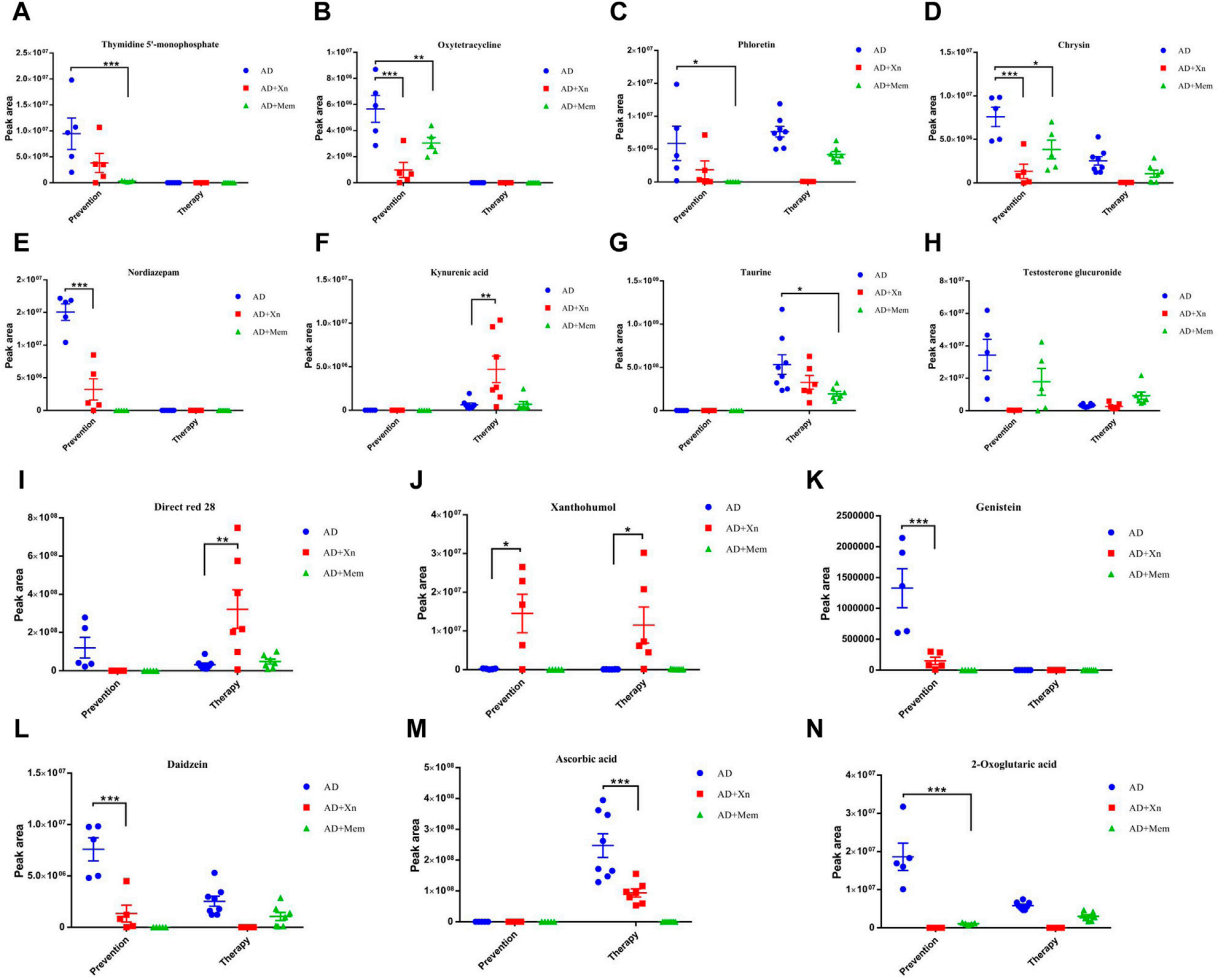
**(A–N)** showed the significant metabolites regulated by preventive and therapeutic treatment of Xn or Mem. n = 5 per group in the prevention experiment. n = 7–8 per group in the therapy experiment. Mem, Memantine. *p < 0.05; **p < 0.01; ***p < 0.001.

To further identify biologically relevant metabolites, KEGG pathway analysis was used to analysis functional associations. In the preventive experiments, we identified 17 significant metabolites that may be involved in 16 KEGG metabolic pathways after Xn treatment of APP/PS1 mice (Supplementary Table S1). The metabolites in these signal pathways were mainly attributed to endogenous metabolites, natural products/medicines, therapeutics/prescription drugs, excipients/additives/colorants and so on. Moreover, some metabolites participate in multiple signaling pathways. We then identified 80 significant metabolites that may be involved in 58 KEGG metabolic pathways after Mem treatment (Supplementary Table S2). Interestingly, four common signal pathways were found between Xn and Mem treatment. The corresponding metabolites were thymidine 5′-monophosphate, oxytetracycline, phloretin, and chrysin, which had the same downregulation effect as Mem (Figures 4A–D). However, the signaling pathway involved with the metabolite nordiazepam in the drugs of abuse/illegal drugs was specifically regulated by Xn and not by Mem (Figure 4E).

In the therapeutic experiments, we identified 15 significant metabolites that may be involved in 21 KEGG metabolic pathways after Xn treatment of APP/PS1 mice (Supplementary Table S3). The metabolites in these signal pathways were mainly attributed to endogenous metabolites, excipients/additives/colorants, extractables/leachables, therapeutics/prescription drugs, and so on. We also identified 14 significant metabolites involved in 24 KEGG metabolic pathways after Mem treatment (Supplementary Table S4). Interestingly, three common signal pathways were found when comparing the effects of Xn and Mem treatment. The corresponding metabolites were kynurenic acid, taurine and testosterone glucuronide (Figures 4F–H). Moreover, the signaling pathway involved in metabolite Direct red 28 in the textile chemicals/auxiliary/dyes was mainly regulated by Xn (Figure 4I).

### 3.4 Identification of potential key metabolites and signal pathway

We also performed a comprehensive comparative analysis of the outcomes of preventive and therapeutic treatments. We found one common KEGG metabolic pathway associated with Xn preventive and therapeutic treatment. The corresponding endogenous metabolite Xn was upregulated in both (Figure 4J). In addition, we found that the metabolic mechanisms of prevention differ from those associated with Xn therapy. The corresponding metabolites oxytetracycline, chrysin, nordiazepam, genistein and daidzein were specifically associated with disease prevention (Figures 4B,D,E,K,L) and testosterone glucuronide, ascorbic acid were specific to disease therapy (Figures 4H,M).

We further found five common KEGG metabolic pathway associated with Mem preventive and therapeutic treatment. The corresponding metabolites phloretin, chrysin, taurine, and 2-oxoglutaric acid were all downregulated (Figures 4C,D,G,N). Testosterone glucuronide was downregulated in the preventive treatment while upregulated after therapeutic treatment (Figure 4H). Notably, phloretin and chrysin were also found in the comparison between Xn and Mem preventive treatment. Taurine was also found in the comparison between Xn and Mem therapeutic treatment. The expression levels were consistent.

We then performed batch color-coding analysis of the screened meaningful signal pathways. Interestingly, the identified significant metabolites Xn, phloretin, chrysin, and apigenin are involved in the flavonoid biosynthesis pathway (Supplementary Figure S3). The identified significant metabolite oxytetracycline participates in the tetracycline biosynthesis pathway downregulated by Xn and Mem preventive treatment (Supplementary Figure S4). The identified key significant metabolites genistein, daidzein, apigenin and glycitein participated in the Isoflavonoid biosynthesis pathway downregulated by both Xn and Mem (Supplementary Figure S5).

## 4 Discussion

Metabolomics has become a popular technique to study AD not only with the goal of discovering potential biomarkers but also identifying novel molecular mechanisms (Horgusluoglu et al., 2022; Amidfar et al., 2024). The metabolome is a collection of small molecular compounds, mainly endogenous small molecules with molecular weights less than 1,000, that are involved in the metabolism of organisms and maintain normal growth function and development. In this study, we first evaluated the neuroprotective effects of Xn from both preventive and therapeutic perspectives in the APP/PS1 double transgenic AD mouse model. We then identified endogenous urinary metabolites and signal pathways related to Xn intervention by applying HPLC-MS/MS analysis and Compound Discoverer (CD) software.

In previous studies (Liu et al., 2022), we found that Xn could improve cognitive function, but whether it was able to reduce anxiety remained unclear. Recent evidences showed that in the early stages of AD, anxiety could arise as an initial compensating behaviour to coping difficulties (Botto et al., 2022). Xn dieted for 8 weeks could play antidepressant-like effects in maternal separation rats (Donoso et al., 2020). However, the present study failed to observe any obvious change in anxiety levels in either 5-month-old or 9-month-old APP/PS1 mice treated with Xn or Mem. This result may be due to factors such as the time, dosage or method of administration.

Evidence of insulin resistance, impaired glucose metabolism, and mitochondrial defects in AD is suggestive of a systematic metabolic disorder (Carvalho et al., 2023; Amidfar et al., 2024). A previous report found significant differences in the urine and fecal metabolic profiles of AD relative to WT mice at 3 and 9 months mice (Zheng et al., 2022). Both cellular and animal experiments have shown that Xn is a protective molecule that improves cognitive function in multiple ways (Huang et al., 2018; Sun et al., 2021; Liu et al., 2022). Here, we found that Xn treatment altered the urinary metabolite profiles at different stages of administration in APP/PS1 mice for the first time. The expression levels of significant metabolites appeared to show an opposite trend, downregulated in the preventive treatment while upregulated in the therapeutic treatment. This was similar to the effects found in mice treated with the positive control drug Mem.

There are many reports on the biomarkers of AD, but metabolic signatures are relatively rare. Previous studies on the mechanism of Xn in protecting the brain or improving cognitive impairment mainly focused on synaptic adjustment, oxidation resistance, anti-inflammatory and anti-apoptosis, among others (Rancán et al., 2017; Sun et al., 2021), rarely on metabolic mechanisms (Kundu et al., 2022). Here, for the first time, we identified potential metabolic biomarkers and explored the mechanism by which Xn improves cognitive dysfunction from the perspective of metabonomics. We first found that nordiazepam and genistein may be important specific metabolic molecules for Xn to prevent cognitive impairment, and they were mainly attributed to the endogenous metabolites, natural products/medicines and therapeutics/prescription drugs, drugs of abuse/illegal drugs, sports doping drugs. Nordiazepam is an intermediate in the hypnotic drug Alprazolam and a ligand for the GABAA receptor benzodiazepine modulatory site. Adverse effects of Alpraxolam are numerous and include memory problems (George et al., 2024). Genistein is a natural isoflavone compound found in legumes and also an estrogen-like compound. As an inhibitor of tyrosine protein kinase, it can improve cognitive impairment by reducing hyperphosphorylation of Tau (Petry et al., 2021), inhibiting neuronal death (Li et al., 2021), and decreasing inflammatory responses and oxidative stress (Shi B et al., 2023), etc. The Isoflavonoid biosynthesis pathway in which genistein participated played a potential biological role in preventing and slowing down cognitive dysfunction of APP/PS1 mice. This may be an important reason why flavonoids can be used as preventive drugs or dietary supplements.

We also found that the metabolic mechanisms of therapeutic treatment of cognitive decline in APP/PS1 mice treated with Xn (9-month-old mice) were different from that used for disease prevention in 3-month-old animals. We found that ascorbic acid (vitamin C) may be important specific metabolic molecule for Xn to treat cognitive impairment. Ascorbic acid is a water-soluble dietary supplement and also essential for many metabolic processes, such as protecting cells from free-radical damage and promoting iron absorption. Ascorbic acid demonstrated antioxidant activity that may be of some benefit for reducing the risk of developing cognitive decline (Bowman, 2012; Harrison et al., 2014; Delrobaei et al., 2019; Clergue-Duval et al., 2021). An endogenous metabolite, Xn, was found both upregulated between Xn preventive and therapeutic treatment, because Xn could be excreted through urine in the form of prototype (Bai et al., 2022). A new metabolite, 2-oxoglutaric acid, was found to be both downregulated between preventive and therapeutic treatment with Mem, an observation worth of further research attention. Although potential differential metabolites were found by CD software, the identified metabolites need further validation because of the complexity and diversity of metabolomics (Cui et al., 2018; Chaleckis et al., 2019).

## 5 Conclusion

We performed a comprehensive non-targeted metabolomic analysis to characterize the urinary metabolite markers of Xn vs Mem treatment in APP/PS1 mice. The results showed that therapeutic administration of Xn could significantly improve cognitive function but not prevent the development of AD-related brain disease. Hundreds of metabolite molecules changed in the urine, and the metabolic profiling varied at different stages of Xn and Mem treatment. This study, for the first time, uncovered signature metabolic profiles and mechanisms of Xn promoting the improvement of cognitive dysfunction from the perspective of metabonomics. These findings help pave a way for developing new therapeutic targets, and evaluating both the clinical efficacy and course of treatment in AD.

## Data Availability

The data presented in this study are available on request from the corresponding author.
