# Supplementary material for: Protective signature of xanthohumol on cognitive function of APP/PS1 mice: a urine metabolomics approach by age
Source: Front Pharmacol. 2024 Jul 24;15:1423060. doi: 10.3389/fphar.2024.1423060 (PMC11303171; doi:10.3389/fphar.2024.1423060)
Supplement: Supplementary file 1 [file DataSheet1.docx]

**Supplementary Materials**

**Protective mechanism of xanthohumol on cognitive function in APP/PS1 mice: a urine metabolomics approach with respect to animal age**

**Contents**

**Supplementary Figure 1** The open field test (OFT) results in APP/PS1 mice treated with Xn or memantine.

**Supplementary Figure 2** Volcano plot analysis of statistically significant compounds in different groups.

**Supplementary Figure 3** Flavonoid biosynthesis pathway regulated by Xn treatment.

**Supplementary Figure 4** Representative of the tetracycline biosynthesis pathway regulated by Xn preventive treatment.

**Supplementary Figure 5** Representative of isoflavonoid biosynthesis pathway regulated by Xn preventive treatment.

**Supplementary Table 1** KEGG pathways analysis of the significant metabolites induced by Xn prophylactic treatment in APP/PS1 mice compared with the control group.

**Supplementary Table 2** KEGG pathways analysis of the significant metabolites induced by memantine prophylactic treatment in APP/PS1 mice compared with the control group.

**Supplementary Table 3** KEGG pathways analysis of the significant metabolites induced by Xn therapeutic treatment in APP/PS1 mice compared with the control group.

**Supplementary Table 4** KEGG pathways analysis of the significant metabolites induced by memantine therapeutic treatment in APP/PS1 mice compared with the control group.


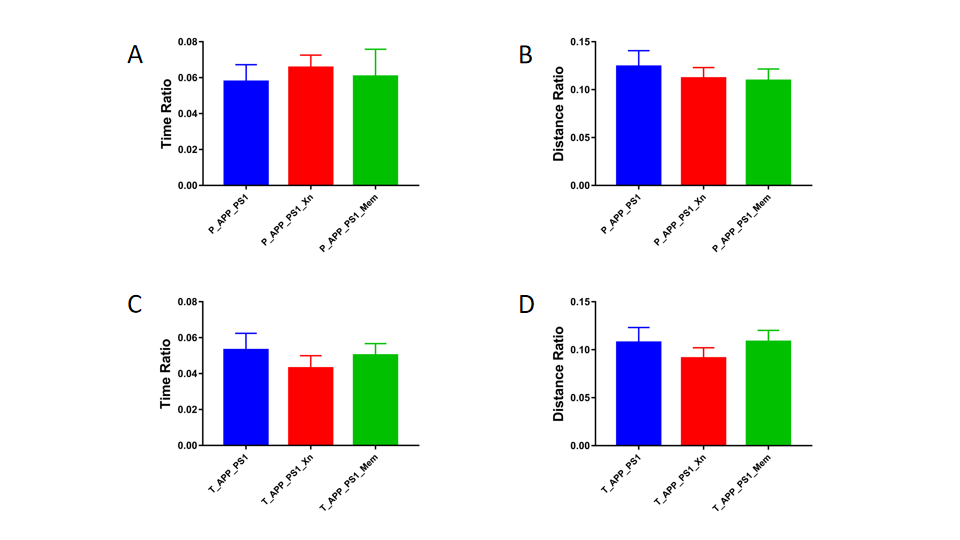


**Supplementary Figure 1** The open field test (OFT) results in APP/PS1 mice treated with Xn or memantine. (A) and (B) showed the time ratio and distance ratio after Xn and memantine treatment in the prevention experiment, respectively. (n=7-11 per group). (C) and (D) showed the time ratio and distance ratio after Xn and memantine treatment in the therapeutic treatment, respectively. (n=10-14 per group). Error bar, standard error of mean (SEM). Mem, memantine.


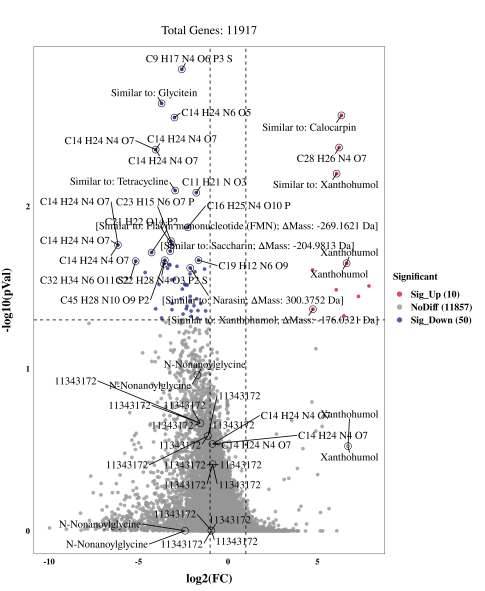

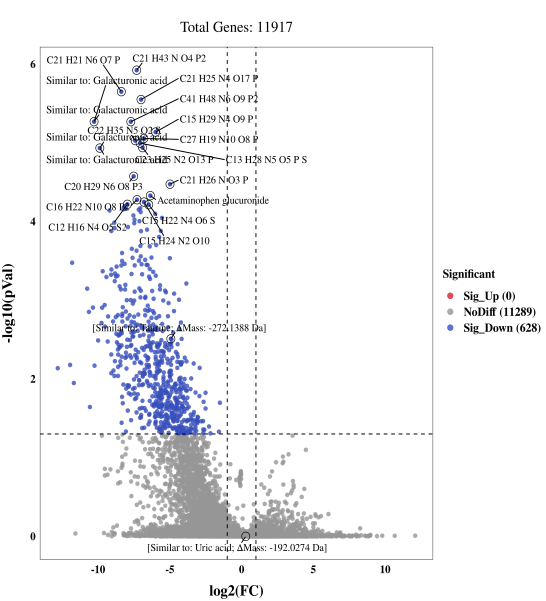

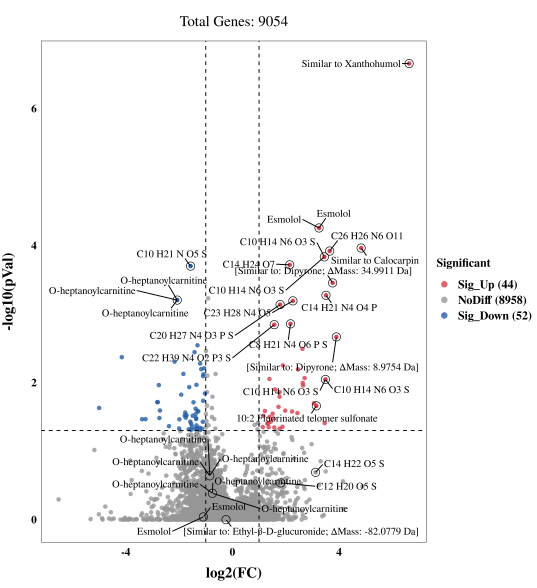

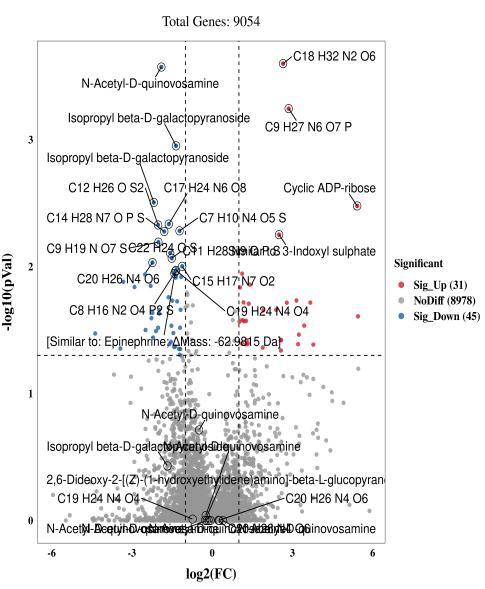


**D**

**C**

**A**

**B**

**Supplementary Figure 2** Volcano plot analysis of statistically significant compounds in different groups.

(A) and (B) showed the comparison results of P_APP_PS1_5Xn vs. P_APP_PS1 and P_APP_PS1_Mem vs. P_APP_PS1, respectively. (C) and (D) showed the comparison results of T_APP_PS1_5Xn vs. T_APP_PS1 and T_APP_PS1_Mem vs. T_APP_PS1, respectively. The x-axis is Log2 fold change while the y-axis is the -Log10 *P* value. Setting parameters: *P* value <0.05, Log2 fold change ≥2. Each data point on the graph represents a compound. The red points on the right represented significantly up-regulated metabolites, while the blue points on the left represented significantly down-regulated metabolites.


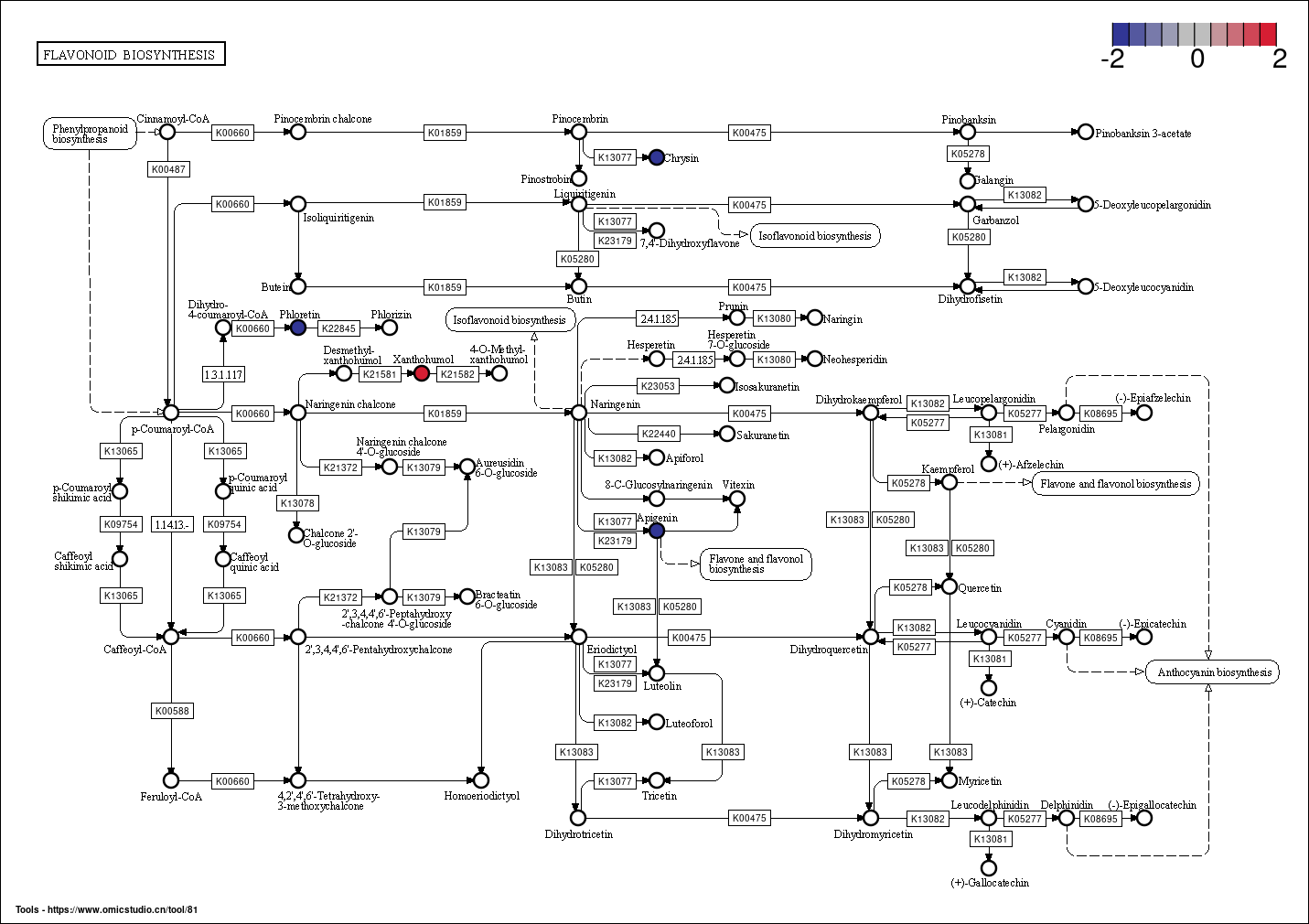


**Supplementary Figure 3** Flavonoid biosynthesis pathway regulated by Xn treatment.


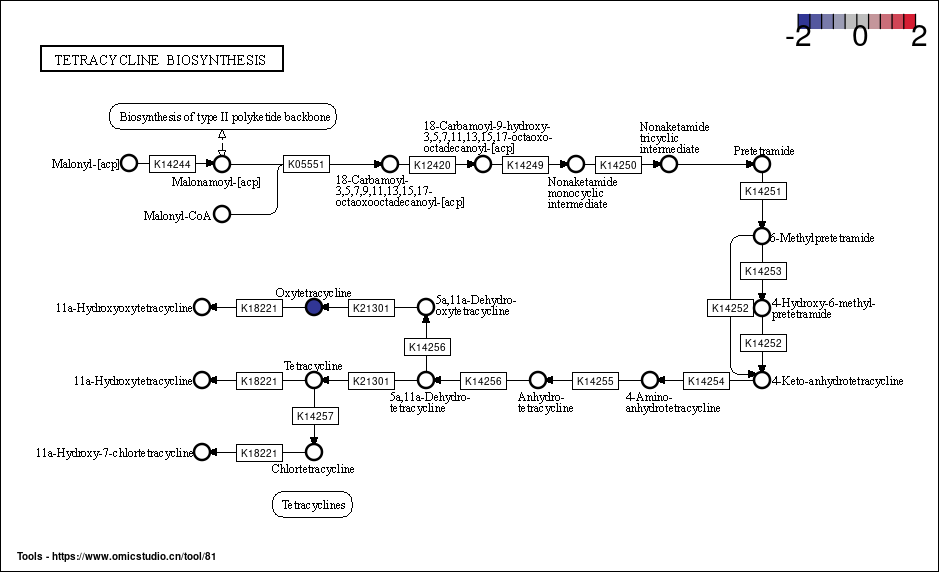


**Supplementary Figure 4** Representative of the tetracycline biosynthesis pathway regulated by Xn preventive treatment.


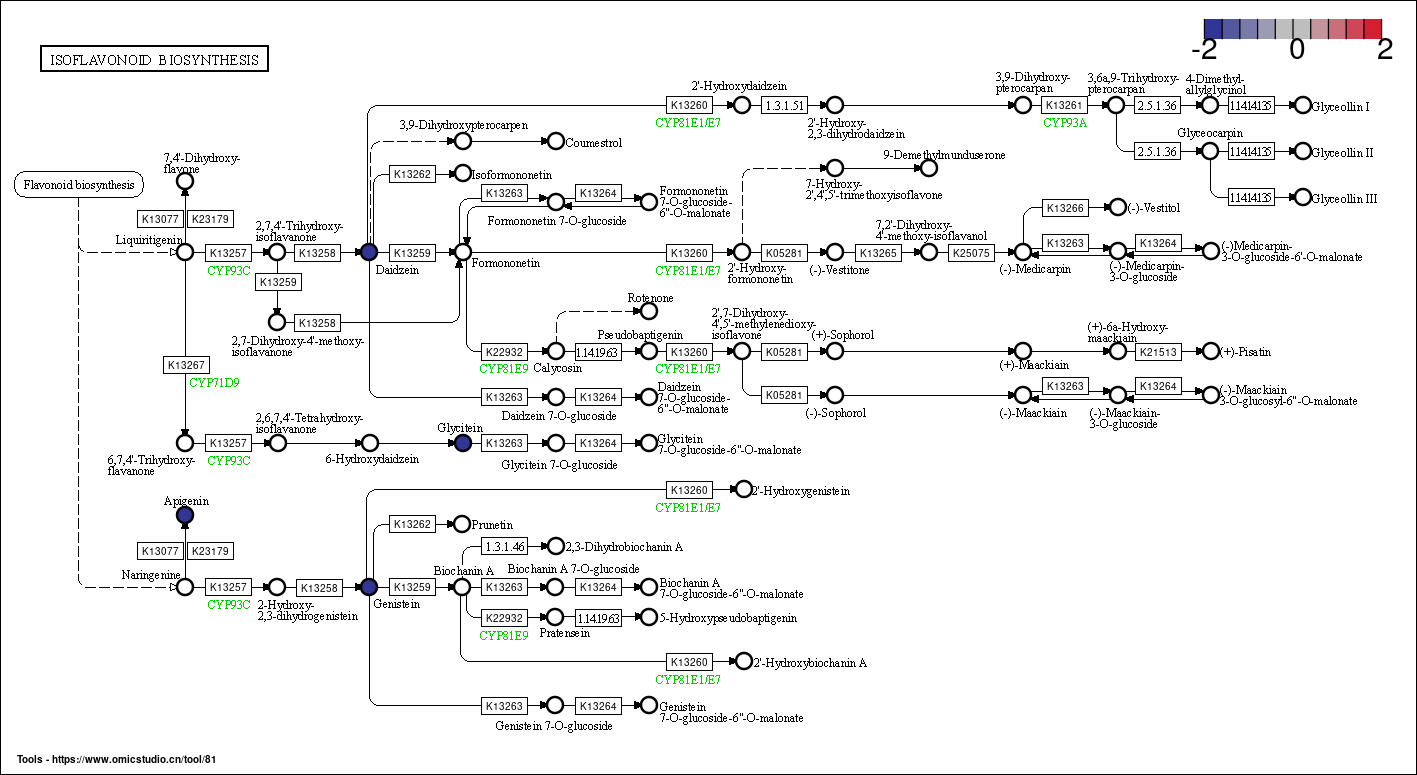


**Supplementary Figure 5** Representative of isoflavonoid biosynthesis pathway regulated by Xn preventive treatment.

**Supplementary Table 1** KEGG pathways analysis of the significant metabolites induced by Xn prophylactic treatment in APP/PS1 mice compared with the control group.

| **KEGG ID** | **Metabolites** | **Formula** | **Molecular weight** | **P value** | **Fold change** | **Trend** |
| --- | --- | --- | --- | --- | --- | --- |
| **Endogenous Metabolites** | | | | | | |
| C00061 | Flavin mononucleotide (FMN) | C17 H21 N4 O9 P | 456.10 | 0.0192 | 0.052 | -4.27 |
| C00364 **†** | Thymidine 5'-monophosphate | C10 H15 N2 O8 P | 322.06 | 0.0301 | 0.187 | -2.42 |
| C00774 **†** | Phloretin | C15 H14 O5 | 274.08 | 0.0358 | 0.232 | -2.11 |
| C01477 | Apigenin | C15 H10 O5 | 270.05 | 0.0260 | 0.230 | -2.12 |
| C14536 | Glycitein | C16 H12 O5 | 284.07 | 0.0023 | 0.076 | -3.71 |
| C16417 **§** | Xanthohumol | C21 H22 O5 | 354.15 | 0.0063 | 67.014 | 6.07 |
| **Endogenous Metabolites; Natural Products/Medicines** | | | | | | |
| C06563 | Genistein | C15 H10 O5 | 270.05 | 0.0260 | 0.230 | -2.12 |
| C10028 **†** | Chrysin | C15 H10 O4 | 254.06 | 0.0240 | 0.097 | -3.37 |
| C10208 | Daidzein | C15 H10 O4 | 254.06 | 0.0240 | 0.097 | -3.37 |
| **Excipients/Additives/Colorants** | | | | | | |
| C02824 | Cyclamic acid | C6 H13 N O3 S | 179.06 | 0.0370 | 0.163 | -2.62 |
| **Therapeutics/Prescription Drugs** | | | | | | |
| D05122 | Narasin | C43 H72 O11 | 764.51 | 0.0014 | 0.167 | -2.58 |
| **Therapeutics/Prescription Drugs; Drugs of Abuse/Illegal Drugs; Sports Doping Drugs; Endogenous Metabolites** | | | | | | |
| D08283 | Nordiazepam | C15 H11 Cl N2 O | 270.06 | 0.0260 | 0.230 | -2.12 |
| **Therapeutics/Prescription Drugs; Endogenous Metabolites** | | | | | | |
| D00201 | Tetracycline | C22 H24 N2 O8 | 444.15 | 0.0080 | 0.130 | -2.95 |
| **Therapeutics/Prescription Drugs; Extractables/Leachables; Excipients/Additives/Colorants; Personal Care Products/Cosmetics; Textile Chemicals/Auxiliary/Dyes; Industrial Chemicals** | | | | | | |
| C12284 | Saccharin | C7 H5 N O3 S | 183.00 | 0.0188 | 0.107 | -3.23 |
| **Therapeutics/Prescription Drugs; Pesticides/Herbicides** | | | | | | |
| C06624 **†** | Oxytetracycline | C22 H24 N2 O9 | 460.15 | 0.0240 | 0.097 | -3.37 |
| **Others** | | | | | | |
| C10402 | Rubiadin | C15 H10 O4 | 254.06 | 0.0240 | 0.097 | -3.37 |

**†** Common metabolic pathways between Xn and Memantine prophylactic treatment in APP/PS1 mice.

**§** Common metabolic pathways between Xn prophylactic treatment and Xn therapeutic treatment in APP/PS1 mice.

**Supplementary Table 2** KEGG pathways analysis of the significant metabolites induced by memantine prophylactic treatment in APP/PS1 mice compared with the control group.

| **KEGG ID** | **Metabolites** | **Formula** | **Molecular weight** | **P value** | **Fold change** | **Trend** |
| --- | --- | --- | --- | --- | --- | --- |
| **Endogenous Metabolites** | | | | | | |
| C00026 **※** | 2-Oxoglutaric acid | C5 H6 O5 | 146.02 | 0.0478 | 0.067 | -3.90 |
| C00089 | Sucrose | C12 H22 O11 | 342.12 | 0.0002 | 0.005 | -7.65 |
| C00232 | Succinic semialdehyde | C4 H6 O3 | 102.03 | 0.0478 | 0.067 | -3.90 |
| C00364 **†** | Thymidine 5'-monophosphate | C10 H15 N2 O8 P | 322.06 | 0.0071 | 0.011 | -6.57 |
| C00366 | Uric acid | C5 H4 N4 O3 | 168.03 | 0.0106 | 0.010 | -6.60 |
| C00581 | Guanidineacetic acid | C3 H7 N3 O2 | 117.05 | 0.0165 | 0.131 | -2.94 |
| C00642 | 4-Hydroxyphenylacetic acid | C8 H8 O3 | 152.05 | 0.0046 | 0.004 | -8.06 |
| C00774 **† ※** | Phloretin | C15 H14 O5 | 274.08 | 0.0223 | 0.015 | -6.11 |
| C01179 | 4-Hydroxyphenylpyruvic acid | C9 H8 O4 | 180.04 | 0.0015 | 0.007 | -7.20 |
| C01197 | Caffeic acid | C9 H8 O4 | 180.04 | 0.0015 | 0.007 | -7.20 |
| C01744 | 3-(4-Hydroxyphenyl)propionic acid | C9 H10 O3 | 166.06 | 0.0134 | 0.049 | -4.34 |
| C01877 | 4-Oxoproline | C5 H7 N O3 | 129.04 | 0.0208 | 0.017 | -5.91 |
| C02519 | 4-Anisic acid | C8 H8 O3 | 152.05 | 0.0046 | 0.004 | -8.06 |
| C05593 | 3-Hydroxyphenylacetic acid | C8 H8 O3 | 152.05 | 0.0046 | 0.004 | -8.06 |
| C05598 | Phenylacetylglycine | C10 H11 N O3 | 193.07 | 0.0165 | 0.131 | -2.94 |
| C05607 | 3-Phenyllactic acid | C9 H10 O3 | 166.06 | 0.0168 | 0.051 | -4.30 |
| C05607 | D(+)-Phenyllactic acid | C9 H10 O3 | 166.06 | 0.0168 | 0.051 | -4.30 |
| C05852 | 2-Hydroxyphenylacetic acid | C8 H8 O3 | 152.05 | 0.0046 | 0.004 | -8.06 |
| C08350 | β-D-Glucopyranuronic acid | C6 H10 O7 | 194.04 | 0.0005 | 0.011 | -6.45 |
| C11237 | β-Estradiol-17β-glucuronide | C24 H32 O8 | 448.21 | 0.0494 | 0.048 | -4.37 |
| C11584 | 4-Methylumbelliferyl glucuronide | C16 H16 O9 | 352.08 | 0.0000 | 0.012 | -6.36 |
| C12270 | N-Acetyl-1-aspartylglutamic acid | C11 H16 N2 O8 | 304.09 | 0.0208 | 0.017 | -5.91 |
| D00643 | Galacturonic acid | C6 H10 O7 | 194.04 | 0.0005 | 0.011 | -6.45 |
| D00856 | DL-Mandelic acid | C8 H8 O3 | 152.05 | 0.0046 | 0.004 | -8.06 |
| D03568 | N-Acetylglycine | C4 H7 N O3 | 117.04 | 0.0016 | 0.006 | -7.49 |
| **Endogenous Metabolites; Excipients/Additives/Colorants** | | | | | | |
| C00109 | 2-Oxobutyric acid | C4 H6 O3 | 102.03 | 0.0478 | 0.067 | -3.90 |
| D01800 | Glucuronic acid-3,6-lactone | C6 H8 O6 | 176.03 | 0.0012 | 0.003 | -8.28 |
| **Endogenous Metabolites; Excipients/Additives/Colorants; Industrial Chemicals** | | | | | | |
| C08261 | Azelaic acid | C9 H16 O4 | 188.10 | 0.0346 | 0.012 | -6.41 |
| **Endogenous Metabolites; Excipients/Additives/Colorants; Personal Care Products/Cosmetics; Industrial Chemicals** | | | | | | |
| D00091 | Vanillin | C8 H8 O3 | 152.05 | 0.0046 | 0.004 | -8.06 |
| **Endogenous Metabolites; Extractables/Leachables; Excipients/Additives/Colorants; Personal Care Products/Cosmetics; Industrial Chemicals; Textile Chemicals/Auxiliary/Dyes** | | | | | | |
| C17714 | Heptanoic acid | C7 H14 O2 | 130.10 | 0.0025 | 0.003 | -8.39 |
| **Endogenous Metabolites; Natural Products/Medicines** | | | | | | |
| C10028 **† ※** | Chrysin | C15 H10 O4 | 254.06 | 0.0430 | 0.082 | -3.61 |
| **Endogenous Metabolites; Personal Care Products/Cosmetics; Excipients/Additives/Colorants** | | | | | | |
| C00245 **※** | Taurine | C2 H7 N O3 S | 125.01 | 0.0031 | 0.033 | -4.93 |
| **Extractables/Leachables; Excipients/Additives/Colorants; Industrial Chemicals; Endogenous Metabolites** | | | | | | |
| C01771 | Crotonic acid | C4 H6 O2 | 86.04 | 0.0037 | 0.018 | -5.83 |
| **Extractables/Leachables; Excipients/Additives/Colorants; Textile Chemicals/Auxiliary/Dyes** | | | | | | |
| C14078 | Direct red 28 | C32 H24 N6 O6 S2 | 652.12 | 0.0001 | 0.002 | -9.20 |
| **Extractables/Leachables; Personal Care Products/Cosmetics; Therapeutics/Prescription Drugs; Natural Products/Medicines; Excipients/Additives/Colorants; Industrial Chemicals; Textile Chemicals/Auxiliary/Dyes** | | | | | | |
| C01432 | DL-Lactic Acid | C3 H6 O3 | 90.03 | 0.0102 | 0.006 | -7.50 |
| **Extractables/Leachables; Textile Chemicals/Auxiliary/Dyes; Perfluorinated Hydrocarbons** | | | | | | |
| C18142 | Perfluoro-1-octanesulfonic acid (PFOS) | C8 H F17 O3 S | 499.94 | 0.0022 | 0.001 | -9.52 |
| **Industrial Chemicals; Excipients/Additives/Colorants** | | | | | | |
| C06104 | Adipic acid | C6 H10 O4 | 146.06 | 0.0354 | 0.024 | -5.38 |
| **Personal Care Products/Cosmetics** | | | | | | |
| C08031 | Dodecyl sulfate | C12 H26 O4 S | 266.16 | 0.0043 | 0.009 | -6.82 |
| **Personal Care Products/Cosmetics; Therapeutics/Prescription Drugs; Industrial Chemicals** | | | | | | |
| C12064 | Resorcinol monoacetate | C8 H8 O3 | 152.05 | 0.0046 | 0.004 | -8.06 |
| **Pesticides/Herbicides** | | | | | | |
| C18599 | Coumafuryl | C17 H14 O5 | 298.08 | 0.0067 | 0.011 | -6.46 |
| **Therapeutics/Prescription Drugs** | | | | | | |
| C02710 | N-Acetyl-L-leucine | C8 H15 N O3 | 173.11 | 0.0199 | 0.032 | -4.95 |
| C07468 | Candesartan | C24 H20 N6 O3 | 440.16 | 0.0019 | 0.005 | -7.67 |
| C20380 | Mycophenolic acid | C17 H20 O6 | 320.13 | 0.0022 | 0.001 | -9.52 |
| D00298 | Digoxin | C41 H64 O14 | 780.43 | 0.0008 | 0.039 | -4.70 |
| D01271 | Bufexamac | C12 H17 N O3 | 223.12 | 0.0066 | 0.053 | -4.23 |
| D08188 | Dipyrone | C13 H17 N3 O4 S | 311.09 | 0.0037 | 0.005 | -7.68 |
| **Therapeutics/Prescription Drugs; Endogenous Metabolites; Natural Products/Medicines** | | | | | | |
| C06973 | Doxycycline | C22 H24 N2 O8 | 444.15 | 0.0213 | 0.013 | -6.23 |
| **Therapeutics/Prescription Drugs; Endogenous Metabolites; Natural Products/Medicines; Excipients/Additives/Colorants** | | | | | | |
| C01912 | (-)-Erythromycin | C37 H67 N O13 | 733.46 | 0.0008 | 0.039 | -4.70 |
| **Therapeutics/Prescription Drugs; Personal Care Products/Cosmetics; Excipients/Additives/Colorants** | | | | | | |
| D04441 | 4-Hexylresorcinol | C12 H18 O2 | 194.13 | 0.0007 | 0.003 | -8.50 |
| **Therapeutics/Prescription Drugs; Pesticides/Herbicides** | | | | | | |
| C06624 **†** | Oxytetracycline | C22 H24 N2 O9 | 460.15 | 0.0430 | 0.082 | -3.61 |
| **Therapeutics/Prescription Drugs; Sports Doping Drugs** | | | | | | |
| D07348 | Adrafinil | C15 H15 N O3 S | 289.08 | 0.0165 | 0.131 | -2.94 |
| **Therapeutics/Prescription Drugs; Sports Doping Drugs; Steroids/Vitamins/Hormones; Endogenous Metabolites** | | | | | | |
| C11134 **※** | Testosterone glucuronide | C25 H36 O8 | 464.24 | 0.0001 | 0.006 | -7.46 |
| **Others** | | | | | | |
| C09781 | Geniposide | C17 H24 O10 | 388.14 | 0.0478 | 0.067 | -3.90 |
| C11326 | 8-Anilino-1-naphthalenesulfonic acid | C16 H13 N O3 S | 299.06 | 0.0012 | 0.001 | -10.36 |
| C11644 | Lamiide | C17 H26 O12 | 422.14 | 0.0478 | 0.067 | -3.90 |
| C14088 | 3-Methylsalicylic acid | C8 H8 O3 | 152.05 | 0.0046 | 0.004 | -8.06 |
| C14829 | (+/-)12(13)-DiHOME | C18 H34 O4 | 314.25 | 0.0025 | 0.003 | -8.39 |
| C16522 | 11(Z),14(Z),17(Z)-Eicosatrienoic acid | C20 H34 O2 | 306.26 | 0.0259 | 0.022 | -5.53 |

**†** Common metabolic pathways between Xn and Memantine prophylactic treatment in APP/PS1 mice.

**※** Common metabolic pathways between Memantine prophylactic treatment and Memantine therapeutic treatment in APP/PS1 mice.

**Supplementary Table 3** KEGG pathways analysis of the significant metabolites induced by Xn therapeutic treatment in APP/PS1 mice compared with the control group.

| **KEGG ID** | **Metabolites** | **Formula** | **Molecular weight** | **P value** | **Fold change** | **Trend** |
| --- | --- | --- | --- | --- | --- | --- |
| **Endogenous Metabolites** | | | | | | |
| C01179 | 4-Hydroxyphenylpyruvic acid | C9 H8 O4 | 180.04 | 0.0194 | 0.363 | -1.46 |
| C01197 | Caffeic acid | C9 H8 O4 | 180.04 | 0.0194 | 0.363 | -1.46 |
| C01717 **‡** | Kynurenic acid | C10 H7 N O3 | 189.04 | 0.0064 | 5.509 | 2.46 |
| C05926 | Neopterin | C9 H11 N5 O4 | 253.08 | 0.0200 | 8.355 | 3.06 |
| C06313 | Biopterin | C9 H11 N5 O3 | 237.09 | 0.0200 | 8.355 | 3.06 |
| C09931 | Hematoxylin | C16 H14 O6 | 302.08 | 0.0194 | 0.363 | -1.46 |
| C10470 | Isoferulic acid | C10 H10 O4 | 194.06 | 0.0432 | 0.192 | -2.38 |
| C16417 **§** | Xanthohumol | C21 H22 O5 | 354.15 | 0.0000 | 98.881 | 6.63 |
| **Endogenous Metabolites; Excipients/Additives/Colorants** | | | | | | |
| C01494 | Ferulic acid | C10 H10 O4 | 194.06 | 0.0432 | 0.192 | -2.38 |
| D01800 | Glucuronic acid-3,6-lactone | C6 H8 O6 | 176.03 | 0.0494 | 0.433 | -1.21 |
| **Endogenous Metabolites; Excipients/Additives/Colorants; Industrial Chemicals** | | | | | | |
| C08261 | Azelaic acid | C9 H16 O4 | 188.10 | 0.0022 | 14.946 | 3.90 |
| **Endogenous Metabolites; Excipients/Additives/Colorants; Steroids/Vitamins/Hormones** | | | | | | |
| D00018 | Ascorbic acid | C6 H8 O6 | 176.03 | 0.0494 | 0.433 | -1.21 |
| **Endogenous Metabolites; Personal Care Products/Cosmetics; Excipients/Additives/Colorants** | | | | | | |
| C00245 **‡** | Taurine | C2 H7 N O3 S | 125.01 | 0.0419 | 0.536 | -0.90 |
| **Extractables/Leachables** | | | | | | |
| C20340 | Ascorbyl stearate | C24 H42 O7 | 442.29 | 0.0494 | 0.433 | -1.21 |
| D03174 | Bumetrizole | C17 H18 Cl N3 O | 315.11 | 0.0455 | 0.427 | -1.23 |
| **Extractables/Leachables; Excipients/Additives/Colorants; Textile Chemicals/Auxiliary/Dyes** | | | | | | |
| C14078 | Direct red 28 | C32 H24 N6 O6 S2 | 652.12 | 0.0004 | 13.555 | 3.76 |
| **Therapeutics/Prescription Drugs** | | | | | | |
| C07468 | Candesartan | C24 H20 N6 O3 | 440.16 | 0.0126 | 3.031 | 1.60 |
| D08188 | Dipyrone | C13 H17 N3 O4 S | 311.09 | 0.0022 | 14.946 | 3.90 |
| **Therapeutics/Prescription Drugs; Sports Doping Drugs; Steroids/Vitamins/Hormones; Endogenous Metabolites** | | | | | | |
| C11134 **‡** | Testosterone glucuronide | C25 H36 O8 | 464.24 | 0.0318 | 0.586 | -0.77 |
| **Others** | | | | | | |
| C11326 | 8-Anilino-1-naphthalenesulfonic acid | C16 H13 N O3 S | 299.06 | 0.0022 | 14.946 | 3.90 |
| C17147 | Neochlorogenic acid | C16 H18 O9 | 354.10 | 0.0194 | 0.363 | -1.46 |

**‡** Common metabolic pathways between Xn and Memantine therapeutic treatment in APP/PS1 mice.

**§** Common metabolic pathways between Xn prophylactic treatment and Xn therapeutic treatment in APP/PS1 mice.

**Supplementary Table 4** KEGG pathways analysis of the significant metabolites induced by memantine therapeutic treatment in APP/PS1 mice compared with the control group.

| **KEGG ID** | **Metabolites** | **Formula** | **Molecular weight** | **P value** | **Fold change** | **Trend** |
| --- | --- | --- | --- | --- | --- | --- |
| **Endogenous Metabolites** | | | | | | |
| C00026 **※** | 2-Oxoglutaric acid | C5 H6 O5 | 146.02 | 0.0210 | 0.499 | -1.00 |
| C00079 | L-Phenylalanine | C9 H11 N O2 | 165.08 | 0.0109 | 0.385 | -1.38 |
| C00774 **※** | Phloretin | C15 H14 O5 | 274.08 | 0.0203 | 0.530 | -0.92 |
| C01717 **‡** | Kynurenic acid | C10 H7 N O3 | 189.04 | 0.0122 | 0.390 | -1.36 |
| C02040 | 4-Hydroxyindole | C8 H7 N O | 133.05 | 0.0146 | 2.050 | 1.04 |
| C05660 | 5-Methoxyindoleacetic acid | C11 H11 N O3 | 205.07 | 0.0146 | 2.050 | 1.04 |
| C13050 | Cyclic ADP-ribose | C15 H21 N5 O13 P2 | 541.06 | 0.0033 | 42.688 | 5.42 |
| C14536 | Glycitein | C16 H12 O5 | 284.07 | 0.0132 | 0.100 | -3.32 |
| **Endogenous Metabolites; Natural Products/Medicines** | | | | | | |
| C10028 **※** | Chrysin | C15 H10 O4 | 254.06 | 0.0445 | 0.439 | -1.19 |
| C10208 | Daidzein | C15 H10 O4 | 254.06 | 0.0445 | 0.439 | -1.19 |
| **Endogenous Metabolites; Personal Care Products/Cosmetics; Excipients/Additives/Colorants** | | | | | | |
| C00245 **‡ ※** | Taurine | C2 H7 N O3 S | 125.01 | 0.0426 | 0.384 | -1.38 |
| **Extractables/Leachables; Industrial Chemicals** | | | | | | |
| C14225 | Bisphenol B | C16 H18 O2 | 242.13 | 0.0395 | 1.564 | 0.64 |
| **Industrial Chemicals** | | | | | | |
| C14130 | 2-tert-Butylphenol | C10 H14 O | 150.10 | 0.0210 | 0.499 | -1.00 |
| C14139 | 4-sec-Butylphenol | C10 H14 O | 150.10 | 0.0210 | 0.499 | -1.00 |
| C14188 | 3-tert-Butylphenol | C10 H14 O | 150.10 | 0.0210 | 0.499 | -1.00 |
| C14200 | 4-tert-butylphenol | C10 H14 O | 150.10 | 0.0210 | 0.499 | -1.00 |
| **Therapeutics/Prescription Drugs** | | | | | | |
| C15482 | Bacitracin A | C66 H103 N17 O16 S | 1421.75 | 0.0441 | 0.093 | -3.43 |
| D01111 | Nateglinide | C19 H27 N O3 | 317.20 | 0.0109 | 0.385 | -1.38 |
| **Therapeutics/Prescription Drugs; Endogenous Metabolites** | | | | | | |
| C00788 | Epinephrine | C9 H13 N O3 | 183.09 | 0.0109 | 0.385 | -1.38 |
| **Therapeutics/Prescription Drugs; Endogenous Metabolites; Excipients/Additives/Colorants; Personal Care Products/Cosmetics; Industrial Chemicals; Pesticides/Herbicides; Natural Products/Medicines** | | | | | | |
| D01039 | Thymol | C10 H14 O | 150.10 | 0.0210 | 0.499 | -1.00 |
| **Therapeutics/Prescription Drugs; Sports Doping Drugs; Steroids/Vitamins/Hormones; Endogenous Metabolites** | | | | | | |
| C11134 **‡ ※** | Testosterone glucuronide | C25 H36 O8 | 464.24 | 0.0114 | 2.180 | 1.12 |
| **Others** | | | | | | |
| C06674 | 3,4-Dihydroxybenzenesulfonic acid | C6 H6 O5 S | 189.99 | 0.0122 | 0.390 | -1.36 |
| C10402 | Rubiadin | C15 H10 O4 | 254.06 | 0.0445 | 0.439 | -1.19 |
| D08111 | Lercanidipine | C36 H41 N3 O6 | 611.30 | 0.0109 | 0.385 | -1.38 |

**‡** Common metabolic pathways between Xn and Memantine therapeutic treatment in APP/PS1 mice.

**※** Common metabolic pathways between Memantine prophylactic treatment and Memantine therapeutic treatment in APP/PS1 mice.
